# Comparing Different Policy Scenarios to Reduce the Consumption of Ultra-Processed Foods in UK: Impact on Cardiovascular Disease Mortality Using a Modelling Approach

**DOI:** 10.1371/journal.pone.0118353

**Published:** 2015-02-13

**Authors:** Patricia V. L. Moreira, Larissa Galastri Baraldi, Jean-Claude Moubarac, Carlos Augusto Monteiro, Alex Newton, Simon Capewell, Martin O’Flaherty

**Affiliations:** 1 Department of Public Health, University of Liverpool, Liverpool, England; 2 Centre for Epidemiological Studies in Health and Nutrition, University of São Paulo, São Paulo, Brazil; 3 Department of Nutrition, School of Public Health, University of São Paulo, São Paulo, Brazil; Universidad Peruana de Ciencias Aplicadas (UPC), PERU

## Abstract

**Background:**

The global burden of non-communicable diseases partly reflects growing exposure to ultra-processed food products (UPPs). These heavily marketed UPPs are cheap and convenient for consumers and profitable for manufacturers, but contain high levels of salt, fat and sugars. This study aimed to explore the potential mortality reduction associated with future policies for substantially reducing ultra-processed food intake in the UK.

**Methods and Findings:**

We obtained data from the UK Living Cost and Food Survey and from the National Diet and Nutrition Survey. By the NOVA food typology, all food items were categorized into three groups according to the extent of food processing: **Group 1** describes unprocessed/minimally processed foods. **Group 2** comprises processed culinary ingredients. **Group 3** includes all processed or ultra-processed products. Using UK nutrient conversion tables, we estimated the energy and nutrient profile of each food group. We then used the IMPACT Food Policy model to estimate reductions in cardiovascular mortality from improved nutrient intakes reflecting shifts from processed or ultra-processed to unprocessed/minimally processed foods. We then conducted probabilistic sensitivity analyses using Monte Carlo simulation.

**Results:**

Approximately 175,000 cardiovascular disease (CVD) deaths might be expected in 2030 if current mortality patterns persist. However, halving the intake of Group 3 (processed) foods could result in approximately 22,055 fewer CVD related deaths in 2030 (*minimum estimate 10,705, maximum estimate 34,625*). An ideal scenario in which salt and fat intakes are reduced to the low levels observed in Group 1 and 2 could lead to approximately 14,235 (*minimum estimate 6,680, maximum estimate 22,525*) fewer coronary deaths and approximately 7,820 (*minimum estimate 4,025, maximum estimate 12,100*) fewer stroke deaths, comprising almost 13% mortality reduction.

**Conclusions:**

This study shows a substantial potential for reducing the cardiovascular disease burden through a healthier food system. It highlights the crucial importance of implementing healthier UK food policies.

## Introduction

Food processing is defined as all methods and techniques used by the food, drink and associated industries to turn fresh whole foods into food products [1]. **Ultra-processed products** (UPP) are assemblages of industrial ingredients obtained from the extraction, refinement, and transformation of constituents of raw foods with usually little or no whole food [2,3].

In contrast to most fresh foods they are frequently sold as ready-to-eat or ready-to-heat foods. They are durable, easy to transport, and designed to be convenient and highly palatable. In the UK, ultra-processed products are generally cheaper than fresh, minimally processed foods and culinary ingredients used in preparing meals [4].

UPPs are typically energy dense and have a high glycaemic load. Processed and ultra-processed products are generally more sugary, salty, fatty, and energy dense than freshly prepared meals and dishes made from unprocessed or minimally processed food and culinary ingredients [5,6]. The poor nutritional content of these foods coupled with patterns of excessive consumption leads to obesity and related chronic **non-communicable diseases** (NCDs) [7,8].

Moubarac et al. (2013) [4] recently displayed the dominance of ready-to-consume products in the UK diet. National food expenditure surveys showed that 58% of calorific intake came from such products. There appears to be a similar trend in Canada, where 55% of dietary energy came from UPPs [6]. Available evidence suggests that it is the high availability, low cost and extensive marketing of ready-to consume UPPs that result in excess intake [9].

People in middle income countries, such as Brazil, are increasing consumption of UPP and these are displacing freshly made meals and dishes [10]. Cheap and easily available calories from UPPs coupled with increasingly sedentary lifestyles in areas experiencing economic transition are thought to be contributing to rising levels of obesity and diabetes. These middle income countries are also suffering rapidly increased morbidity from NCDs, and hence escalating health care expenditure [11]. Extensive evidence suggests that global exposure to the problems caused by UPPs is contributing to a growing burden of NCDs [4].

In 2008, 63% of the 57 million deaths that occurred globally were due to NCDs. Most of these deaths were due to **cardiovascular diseases** (CVD), which account for 48% of NCD. NCDs and CVD are generally associated with four behavioural risk factors: unhealthy diet, tobacco use, physical inactivity and harmful use of alcohol. In relation to diet, the majority of populations consume much higher than recommended levels of sodium, saturated fat and trans-fatty acids. These are all important determinants of high blood pressure and cardiovascular risk [12].

As the global incidence of NCD continues to grow, it is crucial to curb the increase in UPP intake. The World Health Organization (WHO) has therefore developed European Action Plans for Food and Nutrition Policy with the aim of improving upon existing national policies within its European member states. There is particular emphasis on establishing and implementing food-based dietary guidelines and supporting the healthier composition of UPP. It is suggested that this might be achieved by reducing levels of salt, saturated fats and free sugars, and by eliminating industrially produced trans-fatty acids in the modern European diet [13]. The key target set by the WHO is to reduce NCD deaths by 25% before 2025 [14].

However, there is a current scarcity of literature attempting to quantify the links between consumption of UPPs and the development of chronic diseases. Our paper therefore aims to quantify the consumption of UPP in the UK with the development of CVD. We then estimate the potential impact of interventions aimed at reducing UPP consumption. We explore the hypothesis that a reduction in UPP consumption might substantially decrease UK cardiovascular deaths by 2030.

## Methods

We extended the IMPACT Food Policy Model from previous models [15,16] to estimate the mortality reduction achievable through reducing UPP consumption by 2030.

### Estimating the nutrient content of the UPPs groups

Data used in these models is obtained from the Living Cost and Food Survey (LCF, 2011) and it is publicly available. This survey, which is routinely carried out by the Office for National Statistics, uses a sample of roughly 6,000 households. The LCF allows us to estimate the average quantity of food and drink purchased per person per day. Results of the LCF are also compiled in The Family Food Report (2011) [17].

The first step towards modelling effects of dietary change was to classify all food items into three groups according to the nature, extent and purpose of food processing. Group 3 was divided in Group 3a which describes the ‘Processed Food Products’ and Group 3b for ‘Ultra-processed Products’ according to Monteiro et al. (2012) [18]. Further information on the classification system is included in Table 1 [4,18,19].

**Table 1:** Food Classification according to the nature, extent and purpose of food processing.

**Unprocessed or minimally processed food**	**Group 1(G1)** Unprocessed food products	Vegetables, fruits, grains (cereals), legumes (pulses), nuts, roots, tubers, meats, poultry, fish, milk and plain yogurt.
	**Group 2 (G2)** Minimally processed culinary ingredients	Plant oils; animal fats; sugars and syrups; starches and flours, uncooked ‘raw’ pastas made from flour and water, salt.
**Ready-to-consume products**	**Group 3a (G3a)** Processed food products	Canned or bottled whole vegetables and legumes (pulses) preserved in brine; whole fruits preserved in syrup; tinned fish preserved in oil; some types of processed meat and fish such as ham, bacon, pastrami, smoked fish; and cheese, to which salt is added.
	**Group 3b (G3b)** Ultra-processed products	Burgers, frozen pasta, pizza and pasta dishes, ‘nuggets’ and ‘sticks’, crisps (chips), biscuits (cookies), candies, cereal bars, carbonated and other sugared drinks, and a vast array of snack products.

The LCF uses the official UK nutrients conversion table supplied by the Department of Health to convert data on food intake into quantitative measures of the nutritional content. Data on energy content (in kcal and kJ), saturated fats and sodium is taken directly from the LCF. The value of sodium as given by the LCF was converted into salt for use in the analysis. This was done by multiplying the sodium value by 2.5 [20].

However, the LCF provides no measure of trans-fats content. Thus, the National Diet and Nutrition Survey 2011 (NDN) [21] was used in order to estimate trans-fat intake. Because the NDN categorises food differently to the LCF, it was assumed for the purpose of this model that food in Groups 1, 2 and 3a contain zero trans-fats. Only foods in Group 3b were ascribed trans-fat values according to the NDN.

Therefore the inputs for the model were quantitative measures of energy, saturated fats, trans-fats and salt. Results were stratified by age and gender and are shown in S1–S4 Tables. A detailed description of all inputs, including the β Values for CHD and Stroke in relation to age and gender, are described in the S1 Appendix.

### Modelling the effect of changing UPP consumption patterns

We designed two scenarios in order to model the effect of replacing UPP consumption with healthier options: A) ideal and B) feasible.

In the **ideal scenario**, we assumed that dietary intake of G3a and G3b (‘processed or ultra-processed’) foods is entirely replaced with G1 and G2 (‘unprocessed/minimally processed’) foods. This can be expressed as:

G3 = G1+G22

For the **feasible scenario**, we considered that it will be difficult to entirely avoid some items from the ‘processed’ food group (‘3a’ foods like cheese or canned vegetables preserved with brine); therefore we assumed that only G3b products are replaced with an even makeup of G1, G2 and G3a foods. This can be expressed as:

G3b = G1+G2+G3a3

We estimated the change in nutrient composition by subtracting the nutrient levels at the baseline to the healthier group. Then changes in levels of nutrient intake were translated *into a CHD and stroke mortality reduction using meta-analyses of large cohort studies* (Table 2).

**Table 2:** Food policy scenarios and corresponding meta-analyses used to estimate effects.

**Food/Nutrient**	**IDEAL SCENARIO (complete replacement of processed/ultra-processed foods)**	**FEASIBLE SCENARIO (partial replacement of processed/ultra-processed foods)**	**Meta-analyses**
**Salt intake (g/day)**	Salt (G3a+G3b)—Salt (G1+G2/2)	Salt G3b—Salt (G1+G2+3a/3)	Strazzulo et al. (2009)[22] -17% CVD deaths by reducing 5g/day of Salt
**Saturated Fat (% energy intake/day)**	Sat Fat (G3a+G3b)—Sat Fat (G1+G2/2)	Sat Fat G3b—Sat Fat (G1+G2+G3a/3)	Jakobsen et al. (2009)[23] Replacement of 5% energy of Saturated Fat by PUFAs
**Trans-Fat (% energy intake/day)**	Trans-fat (G3a+G3b)—Trans-fat (G1+G2/2)	Trans-fat G3b—Trans-fat (G1+G2+G3a/3)	Mozaffarian & Clark (2009)[24] -12% CHD risk by replacing 1% of energy from Trans-fat with unsaturated fats reduces

CVD, Cardiovascular disease; CHD, Coronary heart disease; PUFAs, Polyunsaturated fatty acids.

First, we defined 2010 as the base year used for projecting the number of UK deaths from coronary heart disease (CHD) and stroke. To calculate the projected number of UK deaths from CHD and stroke in 2030 this base number was multiplied by the projected demographic change over those 20 years.

The predicted reduction in deaths was calculated by multiplying the number of expected deaths by the estimated mortality reduction caused by changes in intake of each nutrient. Predictions were run twice; firstly, using simple addition and assuming non-cumulative effects of the combination of all nutrients changing at the same time. Secondly, accounting for cumulative effects. The generic equations below show how the cumulative and non-cumulative effects were calculated in the ideal scenario as example.

$$Cumulative\;effect\;\left(ideal\;scenario\right)\;=\;\{1-(1-(\beta \;SatFat\times Sat\;Fat\;G3-\left(SatFat\;G1+SatFat\;G2\;÷2\right)*(1-(\beta \;Salt\times Salt\;G3-\left(Salt\;G1+Salt\;G2\;÷2\right)*(1-(\beta \;Trans\;Fat\;G3\;\times \left(TransFat\;G1+TransFat\;G2\;÷2\right)\}$$

$$Non-cumulative\;effect\;(ideal\;scenario)\;=\;(\beta \;SatFat\;\times Sat\;Fat\;G3-\left(SatFat\;G1+SatFat\;G2÷2)\right)+(\beta \;Salt\times Salt\;3-\left(Salt\;G1+Salt\;G2\;÷2\right))+(\beta \;Trans\;Fat\;G3\times \left(TransFat\;G1+TransFat\;G2÷2\right))$$

The projected reduction in deaths was calculated for CHD and stroke separately. The results were stratified by gender and age group. Values were given in both absolute terms and relative to the original predicted number of deaths. (The S1 Appendix provides details of all the equations).

### Sensitivity analysis

Every model involves uncertainty. To explore the potential effects of reducing consumption of UPP on risk factors and CVD deaths, we performed a probabilistic sensitivity analysis. Simulations were performed using the Monte Carlo methodology. This allowed stochastic variation of parameters based on the sizes of the effects obtained from the literature. Using this technique, we were able to recalculate the model iteratively. Details of the distribution choice can be found in S5 Table. Confidence intervals of 95% were generated for the medians using the bootstrap percentile method. The model simulation was implemented using MS Excel with the addition of the Ersatz package (www.epigear.com).

## Results

### Nutrient levels in different food groups

Table 3 shows the average nutrient intake from each food group for the entire sample population (men and women in all age groups). Weighted averages are given along with ranges.

**Table 3:** Nutrient level in different food groups and weighted average in UK 2011.

	**Unprocessed/minimally processed food**		**Processed/ultra-processed food**	
**Food/Nutrient**	**G1**	**G2**	**G3a**	**G3b**
**Salt intake (g/day)**	0.4–0.7 (WA 0.5)	0.05–0.1 (WA 0.06)	0.8–1.35 (WA 0.91)	3.8–5.05 (WA 4.14)
**Saturated Fat (% energy intake/day)**	2.71–3.08 (WA 2.87)	1.24–2.44 (WA 1.61)	1.66–1.82 (WA 1.74)	7.22–7.67 (WA 7.45)
Trans Fat *(% energy intake/day)	0	0	0	0.66 to 0.78 (WA 0.68)

WA = weighted average

*The values for trans-fat are assumptions based on data collected from the National Diet and Nutrition Survey.

### Expected mortality reduction

**Baseline**. In 2010, 80,570 CHD deaths and 49,370 stroke deaths were reported in the UK [25].

If 2010 age-specific rates persist unchanged, approximately 175,000 CVD deaths might be expected in 2030.

**Ideal Scenario**. If the intakes of salt, saturated fat and trans-fat in Group 3 were reduced to the levels observed in Group 1 and 2, it could lead to approximately 22,055 fewer CVD related deaths in 2030 *(minimum estimate 10,705, maximum estimate 34,625)*. (These figs. assume non-cumulative effects.)

These 22,055 fewer deaths would represent a 13% reduction in CVD mortality.

These 22,055 fewer CVD deaths would comprise approximately 9,420 fewer CHD deaths *(min 4,360, max 14,955)* and some 4,180 fewer stroke deaths in men *(min 2,110, max 6,470)*, and in women, approximately 4,815 fewer CHD deaths *(min 2,320, max 7,570)*, and 3,640 fewer stroke deaths *(min 1,915, max 5,630)*.

**Fig 1:**
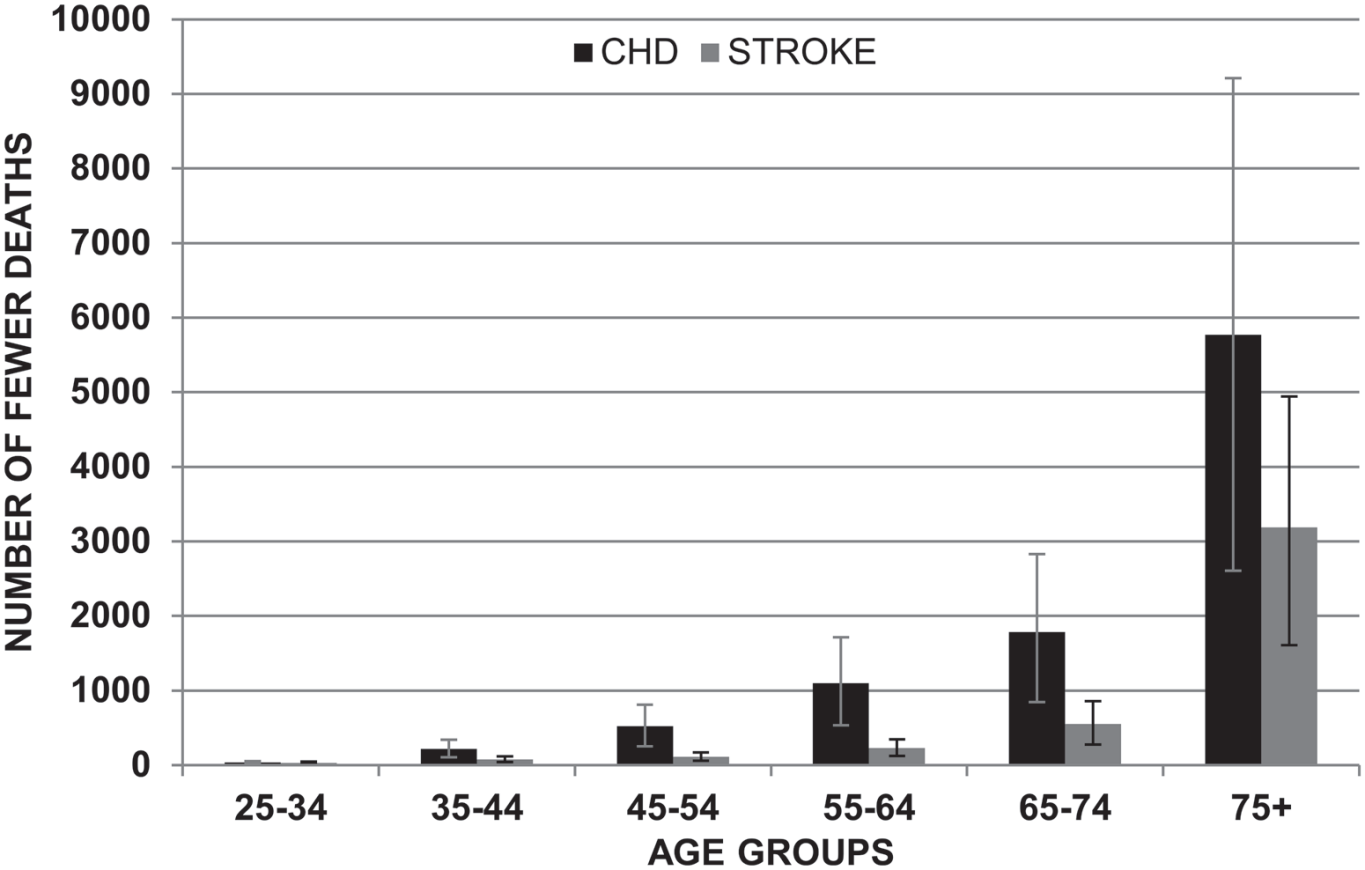
Mean of reduction in CHD and Stroke in males by age group with non-cumulative effects (IDEAL SCENARIO).

**Fig 2:**
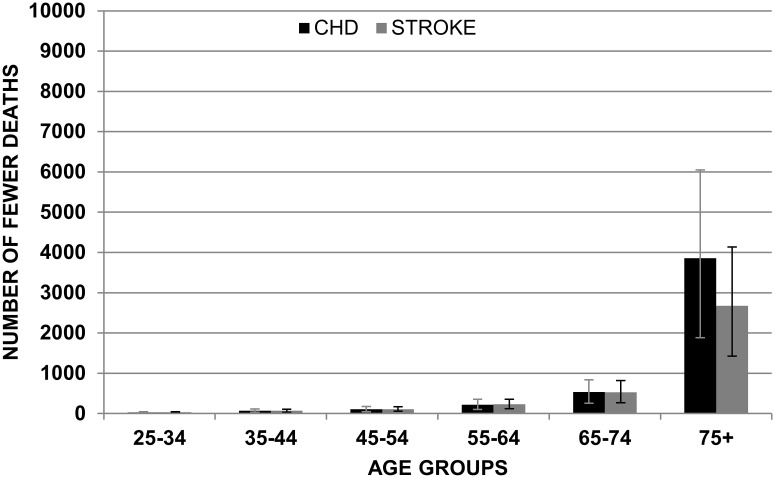
Mean of reduction in CHD and Stroke in females by age group with non-cumulative effects (IDEAL SCENARIO).

Figs. 1 and 2 show the mean reduction in deaths for CHD and stroke in the ‘ideal scenario’ assuming non-cumulative effects.

**Feasible Scenario**. If the intakes of salt, saturated fat and trans-fat from ultra-processed products were reduced to the levels of unprocessed and minimally processed foods and some processed products, it could lead to approximately 17,060 fewer CVD related deaths in 2030 *(minimum estimate 8,145, maximum estimate 27,330)*. This would represent a 10% reduction in CVD mortality.

These 17,060 fewer CVD related deaths would comprise approximately 7,560 fewer CHD deaths *(min 3,375, max 12,300)*, and some 3,010 fewer stroke deaths in men *(min 1,510, max 4,720)*; and in women approximately 3,880 fewer CHD deaths, *(min 1,880, max 6,230)*, and some 2,610 fewer stroke deaths *(min 1,380, max 4,080)*.

The results for both scenarios are illustrated in Table 4. Predictions have been made assuming both cumulative effect and non-cumulative effects.

**Table 4:** Estimated CHD and stroke deaths prevented by achievement of ideal and feasible scenarios in specific food policy options by sex in UK.

**IDEAL SCENARIO**				
	**CHD deaths prevented**		**STROKE deaths prevented**	
	Men deaths prevented (minimum-maximum)	Women deaths prevented (minimum-maximum)	Men deaths prevented (minimum-maximum)	Women deaths prevented (minimum-maximum)
With cumulative effects	9,145 (4,310–14,270)	4,680 (2,300–7,260)	4,110 (2,095–6,320)	3,570 (1,900–5,480)
With non-cumulative effects	9,420 (4,360–14,955)	4,815 (2,320–7,570)	4,180 (2,110–6,470)	3,640 (1,915–5,630)
**FEASIBLE SCENARIO**				
With cumulative effects	7,350 (3,350–11,790)	3,780 (1,860–5,990)	2,950 (1,500–4,590)	2,560 (1,365–3,975)
With non-cumulative effects	7,560 (3,375–12,300)	3,880 (1,880–6,230)	3,010 (1,510–4,720)	2,610 (1,380–4,080)

**Fig 3:**
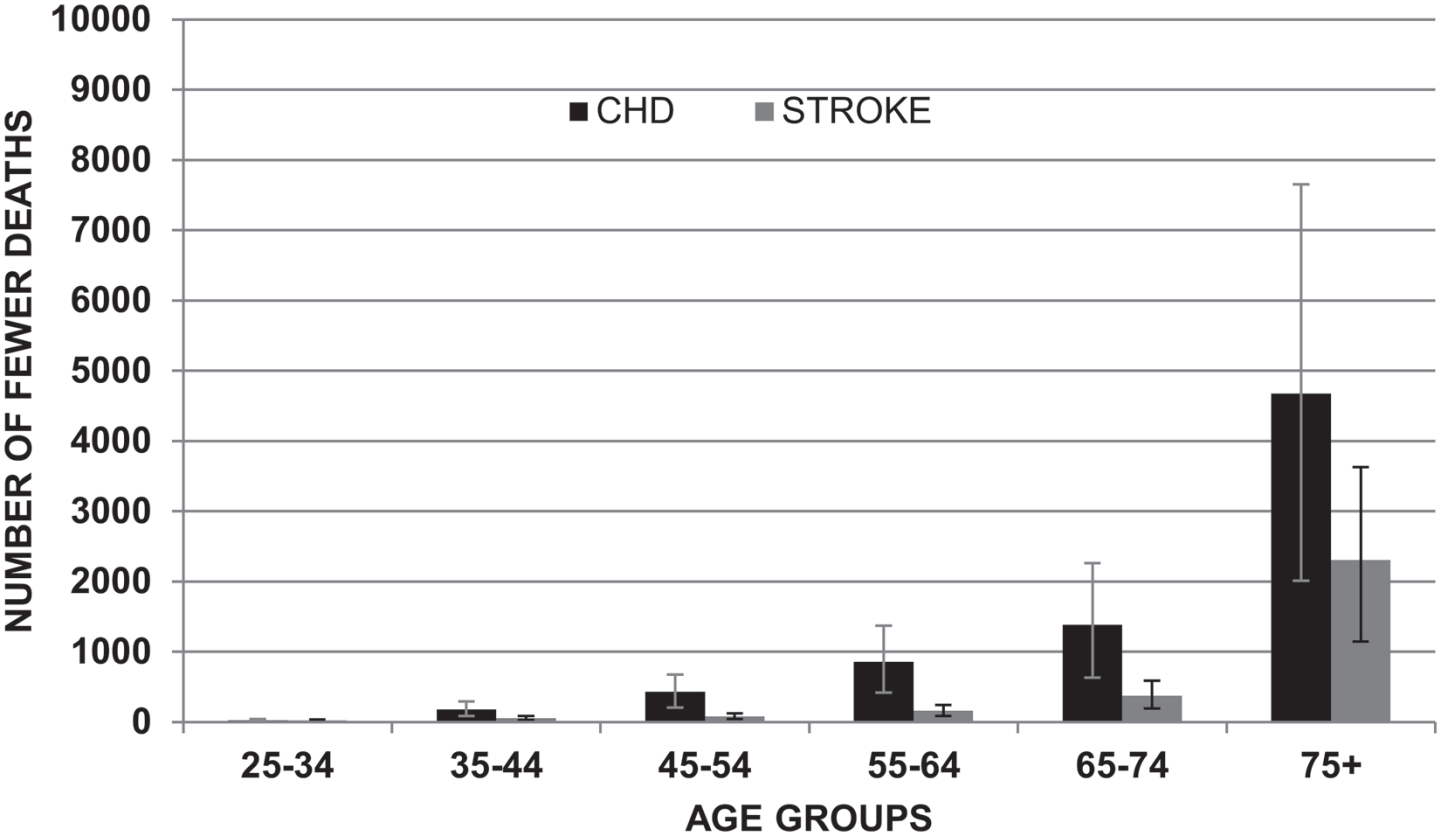
Mean of reduction in CHD and Stroke in males by age group with non-cumulative effects (FEASIBLE SCENARIO).

**Fig 4:**
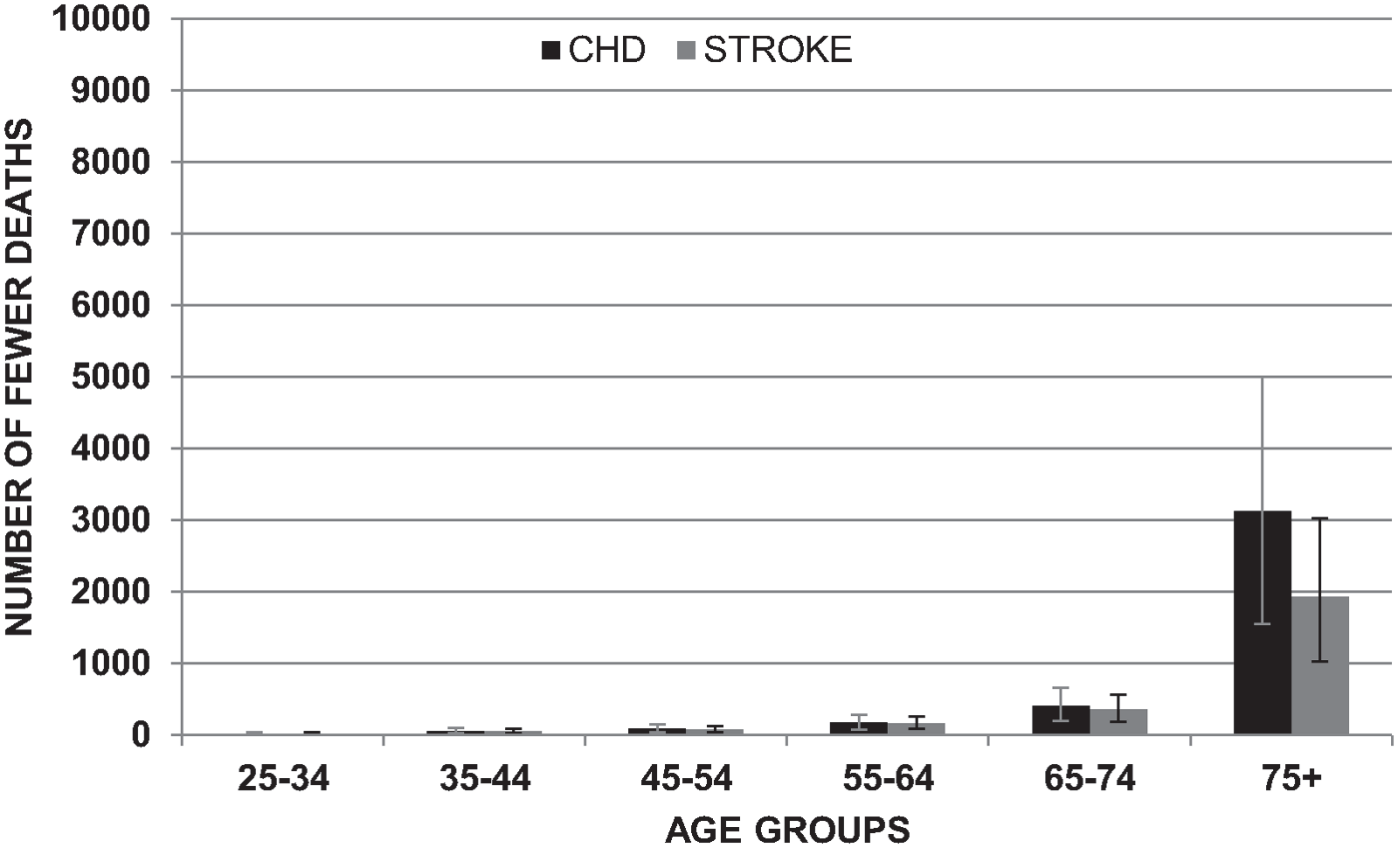
Mean of reduction in CHD and Stroke in females by age group with non-cumulative effects (FEASIBLE SCENARIO).

Figs. 3 and 4 show the mean reduction in deaths for CHD and stroke in the ‘feasible scenario’ assuming non-cumulative effects. Results are subdivided by gender and age group.

## Discussion

The current model was developed to quantify the number of deaths from CHD and stroke that might be prevented by reducing the consumption of processed food products and UPPs in the UK. Both the ideal and feasible scenarios suggest a substantial reduction in CVD mortality, preventing or postponing approximately 17,000 to 22,000 CVD deaths in the UK by 2030.

### Comparison with other modelling studies

Our results are reassuringly consistent with results from other models. A recent analysis in Ireland [16] predicted some 1070 fewer CHD and stroke deaths per year through healthier dietary intakes. To achieve this, the authors proposed increasing fruit and vegetable intake by 3 portions/day and reducing dietary salt by 3 g/day, decreasing trans-fats by 1% of total energy intake and saturated fats by 3% of total energy intake. In our model, our results reflect reductions of around 4 g/day salt, 0.7% total energy for trans-fats, and around 5% lower energy intake saturated fat (Table 3).

A previous UK study suggested that with modest dietary improvements, some 12,000 CVD deaths from could be annually averted. With more substantial improvements this number could increase to around 30,000. This represents around a 20% reduction in CVD mortality [15], slightly more than the 13% in our model.

In the United States, high blood pressure was identified as the main risk factor for CVD mortality, responsible for approximately 395,000 deaths in 2005. High dietary salt, low dietary omega-3 fatty acids, and high dietary trans fatty acids are known to be significant contributors to high blood pressure and were the identified as the dietary risks with the largest mortality effects [26].

The IMPACT models have quantified the contribution of cardiovascular risk factors on CHD mortality trends. Modest reductions in major risk factors such as cholesterol could potentially prevent or postpone approximately 25,000 fewer CHD deaths [27]. Further studies have detected falling CHD mortality related with reductions in risk factors, particularly smoking, cholesterol and blood pressure [28–30].

### Strengths and limitations

This is the first study to explore the potential impact of reducing UPP participation in diet in the UK. We based our effect estimates on high quality nutrition surveys in the UK. Effect sizes were obtained from recent, large meta-analyses and systematic reviews. We also considered that risk factor effects might not be independent and additive, and we therefore explored potential cumulative effects. We also accounted for substantial parameter uncertainty using comprehensive and rigorous probabilistic sensitivity analyses.

However, as in any modelling exercise, our study also has some limitations. Firstly, the sodium intakes in the LCF survey exclude sodium from table salt and they are therefore likely to underestimate the true values. Thus, the average adult dietary salt intake in the UK was estimated to be 8.6 g per day in 2008 falling to 8.1g per day in 2011, (9.3g/d for men and 6.8g/d for women aged 19 to 64 years) [31,32]. However, if the true reduction in salt intake is larger, the number of deaths prevented could be even greater.

Secondly, the LCF survey contains no mention of the quantity of trans-fat in the food. We therefore had to use the National Diet and Nutrition Survey to make assumptions about average intake of trans-fat. However, the trans-fat intake calculated from purchased food is probably not perfectly representative of the true quantity consumed.

Although the LCF survey brings information about expenditures rather than nutrients intakes, studies using Household Budget and Expenditure surveys (HBES) such as the LCF are used to assess dietary practices in over 125 countries. They are generally viewed as being statistically representative, reflecting their large sample size and frequency (being conducted every five years if not annually). However, the mixture of food acquisition and consumption data typically found within HBES can lead to overestimation or underestimation of true consumption [33,34]. Vandevijvere et al., (2013) [34] recommended using HBES in an expanded approach because it is possible to estimate the quantity of UPP consumption in relation to percentage of total calories. However, population individual level consumption data with the same level of detail would be the ideal input for these modelling exercises. Our estimates also do not take into account eat out food and drinks.

We assumed no ‘lag time’ between risk factor change and mortality reduction. Indeed, lag times for CHD are surprisingly short and considerable mortality declines can occur rapidly after individual or population-wide changes in diet [35]. Finally, the model does not track future risk factor trends, and this might result in over or under estimation of the potential reduction. However, given the size of the reductions, the model seems to offer a conservative estimate of potential future gains.

### Public health implications

There is overwhelming evidence that CVD can be powerfully influenced by changes in diet. This is supported by a recent meta-analysis of 8 randomized controlled trials concerning dietary modification [36] and by numerous observational studies [20,37,38].

The high calorific density and excessive quantity of fats, salt and sugars present in UPP make them a danger to cardiovascular health [6,39–41]. Mozaffarian et al. (2010) [36] showed in a meta-analysis that increasing the consumption of polyunsaturated fatty acids as a replacement for saturated fat reduced CHD events by 19%. For every 5% increase in energy derived from PUFA consumption, the risk for CHD reduced by 10%. This study also suggested a reduction in saturated and trans-fat consumption.

High salt consumption is also a powerful determinant of high blood pressure and thus cardiovascular risk. [13]. A modelling study in the United States suggested that if dietary salt intake were reduced by 3g per day, approximately 60,000–120,000 CHD deaths and 32,000–66,000 stroke deaths might be averted annually [42]. In England, the reduction in salt intake from 2003 to 2011 was likely related to a fall in blood pressure and accordingly reduction in CVD [43]. Efforts in salt reduction in the UK, encouraging the industries reformulate the amount of salt in food, resulted in reduction of 0.9 g/day [44] and, in Argentina, a strategy to decrease salt in several food products between 5%–15% of sodium content has the potential to reduce sodium intake by about 400 mg/day [45].

Moubarac et al. (2013) [4] recently showed that the UK diet is over-reliant on ready-to-consume products (G3), either processed or ultra-processed. This is probably due in part to the average cost of ready-to-consume products being preferable in comparison with G1 (unprocessed or minimally processed foods) and G2 (processed culinary ingredients) foods. Having minimal effort of preparation is also seen as advantageous to consumers.

UPP tend to be more palatable and, crucially, affordable to people. They are highly profitable for the food industry. To promote health, the food industry would need to make and market healthier products so as to shift consumption away from ultra-processed, unhealthy foods. However, such foods are intrinsically less profitable than their processed, unhealthy counterparts [46]. In addition, UPPs contribution to ill health goes beyond the nutrients we considered in this study. As important is its contribution to the consumption of excessive levels of energy, mindless eating, giant portions and other mechanisms not captured such as the quantity of free sugars and the low dietary fibre in its content [10].

The contribution of UPP in the diet is set to increase. There is evidence of the progressive industrialization of the food production system increasing the proportion of processed food globally; most notably in lower and middle income countries [5,10,47,48]. Reversing this trend in the UK will not be easy.

However, in some countries such as Brazil, the traditional family meals and dietary pattern of traditional food systems tend to act as protective agents against these ‘villains’. Furthermore, the Brazilian government has supported legislation to protect and improve the traditional food system [49].

However, other countries have demonstrated the practicalities of reducing levels of CHD with dietary changes. In Poland and Finland reducing saturated fat consumption and replacing it in the diet with polyunsaturated fat was shown to dramatically reduce mortality [50,51]. In Poland the socioeconomic transformation after the fall of communism, drastically improved diet quality, resulting in a 40%-fall in CHD mortality, respectively. In Cuba, during the so called “special period”, substantial unplanned but beneficial changes in diet resulted in a dramatic 25% reduction in CHD mortality, along with corresponding decreases in obesity and diabetes. [52]. Such natural experiments demonstrate the potential power of population level changes in diet, as does Finland’s recent successful CVD prevention [51].

Given the extensive market penetration of ultra-processed foods, the task ahead seems impossible. However, evidence is mounting regarding the potential for reduction of the consumption of ultra-processed foods, like sugary drinks taxes [53,54], subsidies to fruits and veg [55,56] and the powerful effect of restriction on marketing food products to children [57].A concerted effort to align powerful, upstream policies targeting the consumption of ultra-processed food and using all public health policy levers is urgently needed.

These results demonstrate the considerable potential for reducing CVD mortality if current trends of consuming UPP are reversed. It is imperative for governments and health authorities to be aware of this information. The general population should also be informed about the harms related to consumption of these products.

## Conclusions

Reducing consumption of processed and ultra-processed products in the UK could result in substantial, 10%–13% decreases in cardiovascular deaths from CVD. This analysis highlights the importance of implementing healthier food UK policies. Although complete elimination of UPPs from the UK diet is unlikely, even halving UPP consumption would still have tangible and powerful public health benefits. Comparable analyses should now be considered in low and middle income countries which are currently experiencing aggressive UPP marketing and rapid dietary transitions.

## Supporting Information

S1 Appendix Detailed description of methods. (DOCX)

S1 Table Salt and Saturated Fat intake by age and gender in Group 1. (DOCX)

S2 Table Salt and Saturated Fat intake by age and gender in Group 2. (DOCX)

S3 Table Salt and Saturated Fat intake by age and gender in group G3a. (DOCX)

S4 Table Salt, Saturated Fat and Trans-Fat intake by age and gender in G3b. (DOCX)

S5 Table Distribution choices for the probabilistic sensitivity analysis. (DOCX)
