# Supplementary material for: Comparing Different Policy Scenarios to Reduce the Consumption of Ultra-Processed Foods in UK: Impact on Cardiovascular Disease Mortality Using a Modelling Approach
Source: PLoS One. 2015 Feb 13;10(2):e0118353. doi: 10.1371/journal.pone.0118353 (PMC4334511; doi:10.1371/journal.pone.0118353)
Supplement: S1 Appendix — (DOCX) [file pone.0118353.s001.docx]

**S1 APPENDIX**

**Detailed Description of Methods**

**Comparing Different Policy Scenarios to Reduce the Consumption of Ultra-Processed Foods in UK: impact on cardiovascular disease mortality using a modelling approach**

Authors: Patricia V. L. Moreira^a^, Larissa Galastri Baraldi^b^, Jean-Claude Moubarac^b^, Carlos Augusto Monteiro^b,c^, Alex Newton^a^, Simon Capewell^a^, Martin O’Flaherty^a^

*^a^ Department of Public Health, University of Liverpool, Liverpool, England*

*^b^ Centre for Epidemiological Studies in Health and Nutrition, University of São Paulo, São Paulo, Brazil*

*^c^ Department of Nutrition, School of Public Health, University of São Paulo, São Paulo, Brazil*

Contents

[List of Abbreviations 2](#_Toc399151481)

[1 INTRODUCTION 3](#_Toc399151482)

[2 METHODS 3](#_Toc399151483)

[2a QUANTIFYING THE AVERAGE NUTRIENT PROFILE OF ULTRA-PROCESSED PRODUCTS AND THE OVERALL DIET IN THE UK 3](#_Toc399151484)

[2a.1 UPP typology description 3](#_Toc399151485)

[2a.2 Data sources and processing 6](#_Toc399151486)

[2a.2.1 Main model Inputs 6](#_Toc399151487)

[2a.2.2 Detailed description of the nutrition related inputs and assumptions 7](#_Toc399151488)

[2b ESTIMATING THE IMPACT OF REDUCING CONSUMPTION OF THESE PRODUCTS UPON MORTALITY FROM CORONARY HEART DISEASE AND STROKE 9](#_Toc399151489)

[2b.1 Simulating the impact of reducing saturated fat, sodium and trans-fat in ultra-processed products and the overall diet 9](#_Toc399151490)

[2b.2. Calculating the Cumulative Mortality Change (CMC) and the Non-cumulative Mortality Change (nCMC) 12](#_Toc399151491)

[2b.3 Estimating the reduction in deaths with cumulative and non-cumulative effects 15](#_Toc399151492)

[2c DESCRIBING THE PROBABILISTIC SENSITIVE ANALYSIS (PSA) 17](#_Toc399151493)

# List of Abbreviations

|  | |
| --- | --- |
| CHD | Cardiovascular heart disease |
| CMC | Cumulative mortality change |
| CVD | Cardiovascular disease |
| DEFRA | Department for Environment, Food and Rural Affairs |
| DH | Department of Health |
| EFS | Expenditure and Food Survey |
| FE | Family Expenditure |
| FSA | Food Standards Agency |
| HNR | Human Nutrition Research |
| IHS | Integrated Household Survey |
| Kcal | Kilo Calorie |
| kJ | Kilo Joules |
| LCFS | Living Costs and Food Survey |
| NFS | National Food Surveys |
| NCD | Noncommunicable diseases |
| Na | Sodium |
| NatCen | NatCen Social Research |
| NCD | Non-communicable diseases |
| ONS | Office for National Statistics |
| SPSS | Statistical Product and Service Solutions |
| UCL | University College London Medical School |
| WHO | World Health Organization |
|  |  |

# 1 INTRODUCTION

In this **Technical Appendix** it will be described the steps to building the modeling. First of all, it is necessary to estimate average nutrient profile and then assess the mortality reduction associated to changes in the average nutrient profile. The survey about food purchased in UK is the Living Cost and Food (2011) and it will be analyzed the nutrient profile of food purchased in overall UK. Then, it will be simulated reductions in the consumption of salt, saturated fat and trans fat and its impacts in reduction CVD deaths.

# 2 METHODS

## 2a QUANTIFYING THE AVERAGE NUTRIENT PROFILE OF ULTRA-PROCESSED PRODUCTS AND THE OVERALL DIET IN THE UK

### 2a.1 UPP typology description

A new food classification based on the nature, extent and purpose of food processing has been devised by Monteiro et al. (2012) at the School of Public Health at the University of Sao Paulo in Brazil [[1](#_ENREF_1),[2](#_ENREF_2)]. The classification categorizes foodstuffs into three main groups. For further information on the classification system please see adapted Box 1 [[3-5](#_ENREF_3)].

**Box 1. Processing methods and examples of foods, ingredients and products.**

| **Group 1: Unprocessed and minimally processed foods**  Minimal processing methods do not add or introduce any new substances to raw foods. Usually minimal processing involves subtracting parts of the food without significantly changing the nature or use of the food. Specific processes include:   - Cleaning, scrubbing, washing - Winnowing, hulling, peeling, flaking, skinning, boning, scaling, filleting, skimming - Drying, chilling, refrigerating, freezing, pasteurization - Sealing, wrapping, vacuum packing   Malting, which involves adding water, is also classed as a minimal process. Fermentation, where living organisms are added, is classed as a minimal process provided it does not generate alcohol.  Minimal processes preserve foods and make them suitable for storage. They ease culinary preparation, can enhance their nutritional quality, make them easier to digest and make them more enjoyable to eat. Unprocessed or minimally processed foods include:   - Fresh, chilled, frozen or vacuum packed vegetables - Fresh fruits, dried fruits and 100% unsweetened fruit juices - Fresh, dried, chilled or frozen meats, poultry and fish - Fresh and pasteurized milk - Eggs - Fermented milk such as plain yoghurt - Grains (cereals) - Fresh, frozen and dried beans and other legumes (pulses) - Roots and tubers - Fungi - Unsalted nuts and seeds - Tea, coffee, herb infusions, tap water and bottled spring water |
| --- |
| **Group 2: Processed culinary ingredients**  In isolation, culinary ingredients are unbalanced, usually being depleted in most nutrients. However, culinary ingredients are normally combined with other foods and are typically not normally consumed by themselves. Specific methods used to produce culinary ingredients include:   - Pressing - Pulverising - Refining - Milling - Crushing - Grinding   These are often used in addition to minimal processing methods. Stabilising or ‘purifying’ agents and other additives may also be used. Examples of ingredients in this category include:   - Plant oils and animal fats such as butter and lard - Sugar - Salt - Starches, flours and uncooked pastas made from oil and water   The function of processed culinary ingredients is to be combined with foods to make palatable, diverse, nourishing and enjoyable dishes and meals. Their nutritional significance should therefore not be assessed in isolation but in combination with whole foods. |
| **Group 3: Ready-to-consume processed products and ultra-processed products**  Group 3a ‘Processed food products’ are directly derived from foods and are recognisable as versions of the original foods. Processed food products are manufactured through processes including:   - Canning and bottling using oils, sugars or salt - Salting, salt pickling - Smoking, curing   Examples include:   - Canned vegetables or legumes preserved in brine - Whole fruits preserved in syrup - Tinned fish preserved in oil - Some processed meats such as ham, bacon, pastrami, smoked fish - Cheese (to which salt is added)   Group 3b, Ultra-processed products, are typically not recognisable as versions of whole foods, although ultra-processing often includes techniques designed to imitate the appearance, shape and sensory qualities of whole foods.  Some are directly derived from the combination of foods and ingredients such as oils, fats, flours, starches, and sugar. Others are made through further processing of food constituents. The majority contain many additives such as preservatives, stabilisers, emulsifiers, solvents, binders, sweeteners, sensory enhancers, flavours and colours. Most of these ingredients used by food manufacturers to make ultra-processed products are not available to consumers. Bulk is often added using air or water. Synthetic micronutrients may be added to ‘fortify’ the products. Examples of group 3b products include:  Pre-prepared meat, fish, vegetable or cheese dishes   - Pre-prepared pizza or pasta dishes - Burgers and hotdogs - Chips and French fries - Poultry and fish ‘nuggets’, ‘sticks’ or ‘fingers’ - Animal products made from flour and salt with scraps/remnants of meat - ‘Instant’ packaged soups and noodles - Bread, breakfast cereals and other cereal products - Sweetened breads and buns - Ice cream, chocolates, confectionery - Cookies, biscuits, cakes, pastries and desserts - Cake mixes - Chips (crisps) and other fatty, salty or sweet snack products - Sugared milk and sweetened fruit drinks - Cola and ‘energy’ drinks - Energy bars - Preserves - Sauces - Meat, yeast and other extracts - Margarines - Canned/dehydrated soups - Instant formula and follow-on milks |

## 2a.2 Data sources and processing

In this section, we present detailed information on model inputs and data sources.

### 2a.2.1 Main model Inputs

#### Table A. Population and data sources used in the IMPACT model

| Information | Source |
| --- | --- |
| Population data |  |
| Population counts by age and sex 2010 and population projection 2030. | SOURCE: 2010-based National Population Projections (*ONS* 2011) |
| Number of deaths by CHD and Stroke | |
| CHD deaths stratified by age and sex  Stroke deaths stratified by age and sex | England and Wales, Office for National Statistics (2010) Deaths registered by cause, sex and age. www.statistics.gov.uk (accessed April 2011). Scotland, General Register Office (2011) Registrar General Annual Report. GRO: Edinburgh. Northern Ireland, Statistics and Research Agency (2011) Report. NISRA: Belfast.  **Coronary heart disease (CHD) - (I20-I25)**  **Stroke - (I60-I69)** |

#### Table B. Data sources for nutrient profile indicators of the food groups

| Information | Source |
| --- | --- |
| Fatty Acids: Saturates (g) UK per day | Living Costs and Food Survey (LCFS) 2011 from household food and drink |
| Na (g) UK per day | Living Costs and Food Survey (LCFS) 2011 from household food and drink |
| Trans Fatty Acids (mean of % food energy) | National Diet and Nutrition Survey (2008/2009 – 2010/11): Average daily intake of energy and macronutrients, by age and sex |

#### Table C. Data sources for effects estimates

| **Information** | **Source** |
| --- | --- |
| **Replacement of 5% energy of SAT FAT by PUFAs** | Jakobsen M. U. et al. (2009) [[6](#_ENREF_6)]. |
|  | |
| **5g change in daily salt intake** | Strazzullo P. et al. (2009) [[7](#_ENREF_7)]. |
| **Replacing 1% of energy from trans-fat with unsaturated fats reduces CHD risk by 12 % (5.5% to 18.5%)** | Mozaffarian D, Clarke R. (2009) [[8](#_ENREF_8)] |

### 2a.2.2 Detailed description of the nutrition related inputs and assumptions

Since 1957 a household expenditure survey has been conducted every year in the UK. Originally data was compiled in the Family Expenditure and National Food Surveys (FES and NFS), which provided information on patterns of household expenditure and food consumption. In April 2001, these surveys were combined to form the Expenditure and Food Survey (EFS). In 2008, selected Government household surveys carried out by the Office for National Statistics (ONS) were combined under the Integrated Household Survey (IHS). At this point the EFS questionnaire became known as the *Living Costs and Food* (LCF).

The ‘household’ is the basic unit of the LCF [[9](#_ENREF_9)]. The survey is voluntary and uses a sample of around 6000 private households. The results from this survey are provided by the Department for Environment, Food and Rural Affairs (DEFRA).

Average food intake per person per day is translated into an average nutritional profile per person per day using the official UK nutrients conversion table. This table uses nutrient composition data supplied by the Department of Health. For further information on nutrient conversion see Family Food 2011 [[9](#_ENREF_9)].

The LCF categorizes food into the following groups:

- Milk and milk products (excluding cheese)
- Cheese
- Carcass meat
- Non-carcass meat and meat products
- Fish
- Eggs
- Fresh and processed fruit and vegetables (including potatoes)
- Sugar and preserves
- Bread
- Flour
- Cakes, buns and pastries
- Biscuits and crisp breads
- Confectionery
- Other cereals and cereal products
- Beverages
- Soft drinks
- Alcoholic drinks
- Other food and drink

All sub items in these groups were transposed into the typology adapted from Group 1 (Unprocessed and minimally processed foods), Group 2 (Processed culinary ingredients), Group 3a (Processed products) or Group 3b (Ultra-processed products) [[4](#_ENREF_4),[5](#_ENREF_5)].

The nutrients examined from LCF data in this model were energy (in Kcal and Joules), saturated fats and sodium. Statistical Product and Service Solutions software (SPSS) was used to calculate the amount of energy, saturated fat and sodium in G1, G2, G3a and G3b, respectively. As salt rather than sodium was used as an input for this model, it was necessary to convert values by multiplying the sodium content by 2.5 [[10](#_ENREF_10)].

The LCF does not provide the quantity of trans-fat in food. In order to estimate the average intake of trans-fat, we used the *National Diet and Nutrition* survey (NDN) [[11](#_ENREF_11)]. The NDN is designed to assess the diet, nutrient intake and nutritional status of the general population (aged 1.5 years and over) living in private households in the UK. The NDN is jointly funded by the DoH in England and the UK Food Standards Agency (FSA). The NDN is carried out by a consortium of three organisations: The National Centre for Social Research (NatCen), MRC Human Nutrition Research (HNR) and the University College London Medical School (UCL) [[11](#_ENREF_11)].

Because the LCF and NDN categorise food differently, it was not possible to calculate the amount of trans-fat in the same way as for sodium and saturated fat. It was assumed that in Groups 1, 2 and 3a the amount of trans-fat would be zero. Only group 3b was assigned values for trans-fat content according to the NDN survey.

## 2b ESTIMATING THE IMPACT OF REDUCING CONSUMPTION OF THESE PRODUCTS UPON MORTALITY FROM CORONARY HEART DISEASE AND STROKE

The next step to take is to quantify the effect of dietary change on average daily nutritional intake in each of the two scenarios run in the model. We can then estimate the subsequent effects on CVD. This is achieved using information from existing studies which quantify such effects.

### 2b.1 Simulating the impact of reducing saturated fat, sodium and trans-fat in ultra-processed products and the overall diet

Figure 1 illustrates relationships between inputs and outputs within the model.

A Δ FOOD GROUP

B Δ NUTRIENT LEVEL

C Δ CORONARY HEART DISEASE AND STROKE MORTALITY

Figure 1: Links in the model

**Scenario 1: ‘Ideal Scenario’**

In the **ideal scenario**, we assumed that dietary intake of G3a and G3b (‘processed and ultra-processed’) foods is entirely replaced with G1 and G2 (‘unprocessed/minimally processed’) foods. This can be expressed as:

$$G3=\frac{\left( G1+G2 \right)}{2}$$

**Scenario 2: ‘Feasible Scenario’**

For the **feasible scenario**, we considered that it will be difficult to avoid entirely some items from the ‘processed’ food group (‘3a’ foods like cheese or canned vegetables preserved with brine), therefore we assumed that only G3b products are replaced with an even makeup of G1, G2 and G3a foods. This can be expressed as:

$$G3b=\frac{\left( G1+G2+G3a \right)}{3}$$

It was necessary for the purposes of analysis to convert saturated fat content from grams into a percentage of total daily energy intakes (Box 2). To achieve this we assumed the following:

**1g Saturated Fat = 9 Kcal [**[**12**](#_ENREF_12)**]**

**Box 2: Conversion from saturated fat to proportion of daily energy intake**

| Fatty Acids: Mean UK intake of Saturates per day (g) and proportion saturates as a percentage of total energy intake amongst individuals under 30 years | | | |
| --- | --- | --- | --- |
| **G1** | **5.29 g** | **5.29*9 = 47.61 Kcal** | **2.94%^(1)^** |
| **G2** | **2.24 g** | **2.24*9 = 20.16 Kcal** | **1.24%^(1)^** |
| **G3a** | **3.10 g** | **3.10*9 = 27.9 Kcal** | **1.72%^(1)^** |
| **G3b** | **13.65 g** | **13.65*9 = 122.85 Kcal** | **7.59%^(1)^** |

^(1)^ In relation to Total Energy = 1618.98 Kcal

* Source: LCF (2011) - values calculated separately by gender and age group

Beta values for CHD and stroke (See Box 3) were taken from a meta-analysis by Jakobsen et al. (2009) [[6](#_ENREF_6)]. These authors proposed replacing 5% of total energy intake coming from saturated fats with energy from polyunsaturated fats and estimated the resulting reduction in cardiovascular mortality.

**Box 3: Beta values for CHD and Stroke in relation to age and sex for Saturated**

| Ages | CHD  Men | STROKE  Men | CHD  Women | STROKE  Women |
| --- | --- | --- | --- | --- |
| 25 to 34 | β = 0.073894737 | β = 0.081710526 | β = 0.073894737 | β = 0.081710526 |
| 35 to 44 | β = 0.073894737 | β = 0.081710526 | β = 0.073894737 | β = 0.081710526 |
| 45 to 54 | β = 0.052 | β = 0.0575 | β = 0.052 | β = 0.0575 |
| 55 - 64 | β = 0.036947368 | β = 0.040855263 | β = 0.036947368 | β = 0.040855263 |
| 65 - 74 | β = 0.027341053 | β = 0.030232895 | β = 0.027341053 | β = 0.030232895 |
| 75-84 | β = 0.026027368 | β = 0.028780263 | β = 0.026027368 | β = 0.028780263 |

* Source: Jakobsen et al. (2009) [[6](#_ENREF_6)]

Salt is preferred to sodium as an input for this model, meaning that we needed to convert our figures from sodium content to salt content. Quantity of salt was calculated using the following assumption:

**1g Salt = 0.4 g sodium [**[**10**](#_ENREF_10)**]**

| Box 4: Conversion of Sodium into Salt | |
| --- | --- |
| Assumption:  1g salt = 0.4g Na | **SALT (g)** |
| G1 | 0.16*1/0.4= 0.4 |
| G2 | 0.02*1/0.4=0.05 |
| G3a | 0.31*1/0.4 = 0.775 |
| G3b | 1.51*1/0.4 = 3.775 |

Example 2:

| Na (g) UK per day age <30 | |
| --- | --- |
| G1 | 0.16 g |
| G2 | 0.02 g |
| G3a | 0.31 g |
| G3b | 1.51 g |

* Source: LCF (2011)

Beta values for the effect of salt on CHD and stroke (See Box 5) were taken from a meta-analysis by Strazzullo et al. (2009) [[13](#_ENREF_13)]. Here the authors demonstrate that reducing salt intake by 5 g/day (equivalent to 2000 mg sodium less per day) translates into approximately 17% fewer CVD deaths each year.

**Box 5: Beta values for CHD and Stroke in relation to age and sex for salt**

| Ages | CHD  Men | STROKE  Men | CHD  Women | STROKE  Women |
| --- | --- | --- | --- | --- |
| 25 to 34 | β = 0.048315789 | β = 0.065368421 | β = 0.048315789 | β = 0.065368421 |
| 35 to 44 | β = 0.048315789 | β = 0.065368421 | β = 0.048315789 | β = 0.065368421 |
| 45 to 54 | β = 0.034 | β = 0.046 | β = 0.034 | β = 0.046 |
| 55 - 64 | β = 0.024157895 | β = 0.032684211 | β = 0.024157895 | β = 0.032684211 |
| 65 - 74 | β = 0.017876842 | β = 0.024186316 | β = 0.017876842 | β = 0.024186316 |
| 75-84 | β = 0.017017895 | β = 0.023024211 | β = 0.017017895 | β = 0.023024211 |

* Source: Strazzullo et al. (2009) [[13](#_ENREF_13)]

The quantity of Trans Fat was provided by *National Diet and Food Survey*. In the Box 6 below, there are the values per age and group of food.

| Box 6:Trans Fatty Acids (mean of % total energy) | |
| --- | --- |
| Men 19-64 | 0.661091084 |
| Men 65 + | 0.779016062 |
| Women 19-64 | 0.67249271 |
| Women 65 + | 0.78601728 |

* SOURCE: National Diet and food survey 2008/09 - 2010/11

A reduction in the consumption of 1.0% of the total energy from trans-fat, according to meta-analysis proposed by Mozaffarian & Clark (2009) [[8](#_ENREF_8)], in which was demonstrated a 12% decrease in CVD deaths for every 1% absolute reduction the consumption of trans-fat (Mozaffarian meta-analysis). From this meta-analysis was extracted the beta values for CHD and Stroke (See Box 7).

**Box 7: Beta values for CHD and Stroke in relation to age and sex for Trans Fat**

| Ages | CHD  Men | STROKE  Men | CHD  Women | STROKE  Women |
| --- | --- | --- | --- | --- |
| 25 to 34 | β = 0.163421053 | β = 0.081710526 | β = 0.163421053 | β = 0.081710526 |
| 35 to 44 | β = 0.163421053 | β = 0.081710526 | β = 0.163421053 | β = 0.081710526 |
| 45 to 54 | β = 0.115 | β = 0.0575 | β = 0.115 | β = 0.0575 |
| 55 - 64 | β = 0.081710526 | β = 0.040855263 | β = 0.081710526 | β = 0.040855263 |
| 65 - 74 | β = 0.060465789 | β = 0.030232895 | β = 0.060465789 | β = 0.030232895 |
| 75-84 | β = 0.057560526 | β = 0.028780263 | β = 0.057560526 | β = 0.028780263 |

* Source: Mozaffarian & Clark (2009)

### 2b.2. Calculating the Cumulative Mortality Change (CMC) and the Non-cumulative Mortality Change (nCMC)

After inputting all β-values into the models, we evaluated the cumulative mortality change (CMC) and the non-cumulative mortality change (nCMC) for CHD and Stroke. The generic equations and examples below show how CMC and nCMC were calculated in both scenarios. For simplicity we have always used the grouping of men under the age of 35 as our examples.

*Cumulative mortality change in the* ***ideal scenario****:*

$$CMC=1-[\left( 1-A \right)\times\left( 1-B \right)\times\left( 1-C \right)]$$

*Where,* ***A=* (βSatFat*SatFatG3-(G1+G2/2),**

**B= (βSalt*SaltG3-(G1+G2/2) and**

**C= (βTransFat*TransFatG3-(G1+G2/2)**

Example: CMC for CHD in men under 35 years in the **ideal scenario**

CMC_1_= 1- {[1-(0.073894737* -0.02505)] * [1-(0.048315789* 0.308333333)] * [1-(0.163421053*0)]}

= 0.013 CMC_1_ for CHD

CMC_2_ = 1- {[1-(0.073894737* 0.03365)] * [1-(0.048315789* 3.304166667)] * [1-(0.163421053*0.6610)]}

= 0.254 CMC_2_ for CHD

CMC for Stroke in men under 35 years of age in the **ideal scenario**

CMC_3_= 1- {[1-(0.018473684* -0.02505)] * [1-(0.065368421* 0.308333333)] * [1-(0.081710526*0)]}

= 0.020 CMC_3_ for Stroke

CMC_4_= 1- {[1-(0.018473684* 0.03365)] * [1-(0.065368421* 3.304166667)] *

[1-(0.081710526*0.6610)]}

= 0.264 CMC_4_ for Stroke

*Cumulative mortality change in the* ***feasible scenario****:*

$$CMC=1-[\left( 1-A \right)\times\left( 1-B \right)\times\left( 1-C \right)]$$

*Where,* ***A=* (βSatFat*SatFatG3b-(G1+G2+G3a/3),**

**B= (βSalt*SaltG3b-(G1+G2+G3a/2) and**

**C= (βTransFat*TransFatG3b-(G1+G2+G3a/3)**

Example: CMC for CHD in men under 35 years in the **feasible scenario**

CMC_1_= 1- {[1-(0.073894737 * 0.076441667)] * [1-(0.048315789* 2.53125)] * [1-(0.163421053* 0.661091084)]}

= 0.223 CMC_1_ for CHD

CMC for Stroke in men under 35 years of age in the **feasible scenario**

CMC_2_= 1- {[1-(0.018473684*0.076441667)] * [1-(0.065368421* 2.53125)] * [1-(0.081710526*0.661091084)]}

= 0.216 CMC_2_ for Stroke

*Non-cumulative mortality change in the* ***ideal scenario****:*

$$nCMC=\left( A+B+C \right)$$

*Where,* ***A=* (βSatFat*SatFatG3-(G1+G2/2),**

**B= (βSalt*SaltG3-(G1+G2/2) and**

**C= (βTransFat*TransFatG3-(G1+G2/2)**

Example: nCMC for CHD in men under 35 years of age in the ideal scenario

nCMC_1_= [(0.073894737* -0.02505) + (0.048315789* 0.308333333) + (0.163421053*0)]

= 0.013 nCMC_1_ for CHD

nCMC_2_= [(0.073894737* 0.03365) + (0.048315789* 3.304166667) + (0.163421053*0.6610)]

= 0.272 nCMC_2_ for CHD

nCMC for Stroke in men under 35 years of age in the ideal scenario

nCMC_3_= [(0.018473684*-0.02505) + (0.065368421*0.308333333) + (0.065368421*0)]

= 0.020 nCMC_3_ for Stroke

nCMC_4_= [(0.018473684*0.03365) + (0.065368421*3.304166667) + (0.065368421*0.6610)]

= 0.276 nCMC_4_ for Stroke

*Non-cumulative mortality change in the* ***feasible scenario****:*

$$nCMC=\left( A+B+C \right)$$

*Where,* ***A=* (βSatFat*SatFatG3b-(G1+G2+G3a/3),**

**B= (βSalt*SaltG3b-(G1+G2+G3a/2) and**

**C= (βTransFat*TransFatG3b-(G1+G2+G3a/3)**

Example: nCMC for CHD in men under 35 years of age in the feasible scenario

nCMC_1_= [(0.073894737* 0.076441667) + (0.048315789* 2.53125) + (0.163421053*0.661091084)]

= 0.237 nCMC_1_ for CHD

nCMC for Stroke in men under 35 years of age in the feasible scenario

nCMC_2_= [(0.018473684*0.076441667) + (0.065368421*2.53125) + (0.065368421*0.661091084)]

= 0.225 nCMC_2_ for Stroke

As there are multiple variables involved in these equations it is important to ensure that the beta value is correct for the relevant age group, gender and outcome (CHD or stroke) that is being calculated. This applies to calculations of both CMC and nCMC.

### 2b.3 Estimating the reduction in deaths with cumulative and non-cumulative effects

Finally, to precisely estimate absolute figures for mortality reduction we multiplied the predicted change (CMC or nCMC) by the projected population in 2030. The projected figures used account for changes in population demographic as well as simply population size. This is important given the distribution of CVD mortality.

Once again, generic formulae are given along with an example. The examples all apply to men under 35 years of age.

*Reduction in CHD mortality using cumulative effects:*

Expected Deaths from CHD*CMC_1_^­^+ Expected Deaths from CHD*CMC_2_

Where: Expected Deaths for CHD = age group CHD mortality rate in 2010*projected population demographics in 2030

Example: Reduction in CHD mortality for men under 35 years of age in the ideal scenario using cumulative effects:

= [(0.00001* 15114438)*0.013] + [(0.00001* 15114438)*0.254] = 30

Example: Reduction in CHD mortality for men under 35 years of age in the feasible scenario using cumulative effects:

= [(0.00001* 15114438)*0.223] = 25

*Reduction in CHD mortality using non-cumulative effects:*

Expected Deaths from CHD*nCMC_1_ + Expected Deaths from CHD*nCMC_2_

Where: Expected Deaths for CHD = age group CHD mortality rate in 2010*projected population demographics in 2030

Example: Reduction in CHD mortality for men under 35 years of age in the ideal scenario using non-cumulative effects

= [(0.00001* 15114438)*0.013] + [(0.00001* 15114438)*0.272] = 32

Example: Reduction in CHD mortality for men under 35 years of age in the feasible scenario using non-cumulative effects

= [(0.00001* 15114438)*0.237] = 26

*Reduction in stroke mortality using non-cumulative effects:*

Expected Deaths from stroke*CMC_1_ + Expected Deaths from stroke*CMC_2_

Where: Expected Deaths for Stroke = age group Stroke mortality rate in 2010* projected population demographics in 2030

Example: Reduction in stroke mortality for men under 35 years of age in the ideal scenario using cumulative effects:

= [(0.00001* 15114438)*0.020] + [(0.00001* 15114438)*0.264] = 28

Example: Reduction in stroke mortality for men under 35 years of age in the ideal scenario using cumulative effects:

= [(0.00001* 15114438)*0.216] = 21

*Reduction in stroke mortality using non-cumulative effects:*

Expected Deaths from stroke*nCMC_3_+ Expected Deaths from stroke*nCMC_4_

Where: Expected Deaths for Stroke = age group Stroke mortality rate in 2010* projected population demographics in 2030

Example: Reduction in stroke mortality for men under 35 years of age in the ideal scenario using non-cumulative effects

= [(0.00001* 15114438)*0.020] + [(0.00001* 15114438)*0.276] = 29

Example: Reduction in stroke mortality for men under 35 years of age in the ideal scenario using non-cumulative effects

= [(0.00001* 15114438)*0.225] = 22

The same reasoning and process is applied to all age groups and both sexes in both scenarios.

The figures derived from the examples given above are shown in the following box (Box 8):

**Box 8: Number of reduction in CHS and Stroke deaths in both scenarios (age group under 35 male)**

| **Age group < 35 Male** | Reduction in CHD deaths | | Reduction in Stroke Deaths | |
| --- | --- | --- | --- | --- |
|  | **Ideal Scenario** | **Feasible scenario** | **Ideal Scenario** | **Feasible scenario** |
| **With cumulative effects** | 30 | 25 | 28 | 21 |
| **With non-cumulative effects** | 32 | 26 | 29 | 22 |

## 2c DESCRIBING THE PROBABILISTIC SENSITIVE ANALYSIS (PSA)

Every model involves uncertainty. To explore the potential effects of reducing consumption of UPP on risk factors and CVD deaths we performed a probabilistic sensitivity analysis. Simulations were performed using the Monte Carlo methodology. This allowed stochastic variation of parameters based on the sizes of the effects obtained from the literature. Using this technique we were able to recalculate the model iteratively. Confidence intervals of 95% were generated for the median using the bootstrap percentile method. The model simulation was implemented using MS Excel with the addition of the package Ersatz ([www.epigear.com](http://www.epigear.com)).

Box 9 shows the distribution of values used for each nutrient/food input.

| Box 9: Standard distribution function used in the model | |
| --- | --- |
| Nutrient/food | **Distribution used** |
| Salt | Erpert *= (lower confidence interval, mean, upper confidence interval) |
| Saturated Fat | Erpert *= (lower confidence interval, mean, upper confidence interval) |
| Trans-fat | Ernormal **= (mean, standard deviation) |
| *Erpert = Pert standard distribution functions  **Ernormal = Normal standard distribution function | |

***Reference list***

1. Monteiro CA, Levy RB, Claro RM, Castro IR, Cannon G (2010) A new classification of foods based on the extent and purpose of their processing. Cad Saude Publica 26: 2039-2049.

2. Monteiro CA, Cannon G (2012) The Impact of Transnational “Big Food” Companies on the South: A View from Brazil. PLoS Med 9: e1001252.

3. Moubarac J-C, Claro RM, Baraldi LG, Levy RB, Martins APB, et al. (2013) International differences in cost and consumption of ready-to-consume food and drink products: United Kingdom and Brazil, 2008–2009. Global Public Health 8: 845-856.

4. Monteiro CA CG, Levy RB, Claro RM, Moubarac J-C. (2012) The Food System. Ultra-processing. The big issue for nutrition, disease, health, well-being.

[Commentary]. World Nutrition Journal of the World Public Health Nutrition Association 3: 527-569.

5. Moubarac J-C, Parra D, Cannon G, Monteiro C (2014) Food Classification Systems Based on Food Processing: Significance and Implications for Policies and Actions: A Systematic Literature Review and Assessment. Current Obesity Reports: 1-17.

6. Jakobsen MU, O'Reilly EJ, Heitmann BL, Pereira MA, Bälter K, et al. (2009) Major types of dietary fat and risk of coronary heart disease: a pooled analysis of 11 cohort studies. The American Journal of Clinical Nutrition 89: 1425-1432.

7. Strazzullo P, D’Elia L, Kandala N-B, Cappuccio FP (2009) Salt intake, stroke, and cardiovascular disease: meta-analysis of prospective studies. BMJ 339.

8. Mozaffarian DC, R (2009) Quantitative effects on cardiovascular risk factors and coronary heart disease risk of replacing partially hydrogenated vegetable oils with other fats and oils. Eur J Clin Nutr 63: S22-S33.

9. Department for Environment FaRA (2012) Family Food 2011.

10. He FJ, MacGregor GA (2010) Reducing Population Salt Intake Worldwide: From Evidence to Implementation. Progress in Cardiovascular Diseases 52: 363-382.

11. Agency DoHatFS (2012) National Diet and Nutrition Survey. Headline results from Years 1, 2 and 3 (combined) of the Rolling Programme (2008/2009 – 2010/11). .

12. Nutrition SACo (2011) Dietary Reference Values for

Energy. .

13. Pasquale S, Lanfranco DE, Ngianga-Bakwin K, Francesco PC (2009) Salt intake, stroke, and cardiovascular disease: meta-analysis of prospective studies. BMJ 339.
