# Supplementary material for: Comparing Different Policy Scenarios to Reduce the Consumption of Ultra-Processed Foods in UK: Impact on Cardiovascular Disease Mortality Using a Modelling Approach
Source: PLoS One. 2015 Feb 13;10(2):e0118353. doi: 10.1371/journal.pone.0118353 (PMC4334511; doi:10.1371/journal.pone.0118353)
Supplement: S1 Table — (DOCX) [file pone.0118353.s002.docx]

**S1Table: Salt and Saturated Fat intake by age and gender in Group 1.**

|  | SALT in Group 1  (g) | | | SAT FAT in Group 1  % of energy | | |
| --- | --- | --- | --- | --- | --- | --- |
| Age and gender | **Mean** | **LIC** | **UIC** | **Mean** | **LIC** | **UIC** |
| 25-34 M* | 0.4 | 0.3 | 0.5 | 0.0294 | 0.0221 | 0.0367 |
| 25-34 F** | 0.4 | 0.3 | 0.5 | 0.0294 | 0.0221 | 0.0367 |
| 35-44 M | 0.45 | 0.375 | 0.525 | 0.0277 | 0.0233 | 0.0322 |
| 35-44 F | 0.45 | 0.375 | 0.525 | 0.0277 | 0.0233 | 0.0322 |
| 45-54 M | 0.475 | 0.4 | 0.725 | 0.0271 | 0.0222 | 0.032 |
| 45-54 F | 0.475 | 0.4 | 0.725 | 0.0271 | 0.0222 | 0.032 |
| 55-64 M | 0.65 | 0.55 | 0.725 | 0.0283 | 0.0241 | 0.0324 |
| 55-64 F | 0.65 | 0.55 | 0.725 | 0.0283 | 0.0241 | 0.0324 |
| 65-74 M | 0.75 | 0.625 | 0.875 | 0.0287 | 0.0237 | 0.0337 |
| 65-74 F | 0.75 | 0.625 | 0.875 | 0.0287 | 0.0237 | 0.0337 |
| 75+ M | 0.7 | 0.575 | 0.825 | 0.0308 | 0.0248 | 0.0369 |
| 75+ F | 0.7 | 0.575 | 0.825 | 0.0308 | 0.0248 | 0.0369 |

***M = male; **F = female (It was assumed the same values for male and female)**
