# Supplementary material for: Comparing Different Policy Scenarios to Reduce the Consumption of Ultra-Processed Foods in UK: Impact on Cardiovascular Disease Mortality Using a Modelling Approach
Source: PLoS One. 2015 Feb 13;10(2):e0118353. doi: 10.1371/journal.pone.0118353 (PMC4334511; doi:10.1371/journal.pone.0118353)
Supplement: S2 Table — (DOCX) [file pone.0118353.s003.docx]

**S2Table: Salt and Saturated Fat intake by age and gender in Group 2.**

|  | **SALT in Group 2**  **(g)** | | | **SAT FAT in Group 2**  **% of energy** | | |
| --- | --- | --- | --- | --- | --- | --- |
| Age and gender | **Mean** | **LIC** | **UIC** | **Mean** | **LIC** | **UIC** |
| 25-34 M* | 0.05 | 0.025 | 0.05 | 0.0124 | 0.0077 | 0.0172 |
| 25-34 F** | 0.05 | 0.025 | 0.05 | 0.0124 | 0.0077 | 0.0172 |
| 35-44 M | 0.05 | 0.05 | 0.075 | 0.0147 | 0.0116 | 0.0178 |
| 35-44 F | 0.05 | 0.05 | 0.075 | 0.0147 | 0.0116 | 0.0178 |
| 45-54 M | 0.05 | 0.05 | 0.075 | 0.0162 | 0.0135 | 0.019 |
| 45-54 F | 0.05 | 0.05 | 0.075 | 0.0162 | 0.0135 | 0.019 |
| 55-64 M | 0.1 | 0.075 | 0.1 | 0.0203 | 0.0174 | 0.0232 |
| 55-64 F | 0.1 | 0.075 | 0.1 | 0.0203 | 0.0174 | 0.0232 |
| 65-74 M | 0.1 | 0.1 | 0.125 | 0.0244 | 0.02 | 0.0286 |
| 65-74 F | 0.1 | 0.1 | 0.125 | 0.0244 | 0.02 | 0.0286 |
| 75+ M | 0.1 | 0.1 | 0.125 | 0.0239 | 0.0195 | 0.0283 |
| 75+ F | 0.1 | 0.1 | 0.125 | 0.0239 | 0.0195 | 0.0283 |

***M = male; **F = female (It was assumed the same values for male and female)**
