# Supplementary material for: Comparing Different Policy Scenarios to Reduce the Consumption of Ultra-Processed Foods in UK: Impact on Cardiovascular Disease Mortality Using a Modelling Approach
Source: PLoS One. 2015 Feb 13;10(2):e0118353. doi: 10.1371/journal.pone.0118353 (PMC4334511; doi:10.1371/journal.pone.0118353)
Supplement: S4 Table — (DOCX) [file pone.0118353.s005.docx]

**S4Table: Salt, Saturated Fat and Trans-Fat intake by age and gender in G3b.**

|  | **SALT (g) in G3b** | | | **SAT FAT (%) in G3b** | | | **TRANS FAT (%) in G3b** | |
| --- | --- | --- | --- | --- | --- | --- | --- | --- |
| **Age and gender** | **Mean** | **LIC** | **UIC** | **Mean** | **LIC** | **UIC** | **Mean of % of energy** | **Standard Deviation** |
| 25-34 M* | 3.775 | 2.925 | 4.625 | 0.0759 | 0.0564 | 0.0954 | 0.661091084 | 0.280594503 |
| 25-34 F** | 3.775 | 2.995 | 4.625 | 0.0759 | 0.0564 | 0.0954 | 0.67249271 | 0.347500005 |
| 35-44 M | 3.825 | 3.25 | 4.4 | 0.0735 | 0.0612 | 0.0857 | 0.661091084 | 0.280594503 |
| 35-44 F | 3.825 | 3.25 | 4.4 | 0.0735 | 0.0612 | 0.0857 | 0.67249271 | 0.347500005 |
| 45-54 M | 4.15 | 3.575 | 4.725 | 0.0735 | 0.0628 | 0.0842 | 0.661091084 | 0.280594503 |
| 45-54 F | 4.15 | 3.575 | 4.725 | 0.0735 | 0.0628 | 0.0842 | 0.67249271 | 0.347500005 |
| 55-64 M | 4.875 | 4.275 | 5.45 | 0.0722 | 0.0627 | 0.0817 | 0.661091084 | 0.280594503 |
| 55-64 F | 4.875 | 7.275 | 5.45 | 0.0722 | 0.0627 | 0.0817 | 0.67249271 | 0.347500005 |
| 65-74 M | 5.05 | 4.15 | 5.95 | 0.0728 | 0.0597 | 0.0859 | 0.779016062 | 0.349748891 |
| 65-74 F | 5.05 | 4.15 | 5.95 | 0.0728 | 0.0597 | 0.0859 | 0.67249271 | 0.297838754 |
| 75+ M | 4.7 | 3.75 | 5.65 | 0.0767 | 0.0615 | 0.0919 | 0.779016062 | 0.349748891 |
| 75+ F | 4.7 | 3.75 | 5.65 | 0.0767 | 0.0615 | 0.0919 | 0.78601728 | 0.297838754 |

***M = male; **F = female (It was assumed the same values for male and female)**
