# Supplementary material for: Comparing Different Policy Scenarios to Reduce the Consumption of Ultra-Processed Foods in UK: Impact on Cardiovascular Disease Mortality Using a Modelling Approach
Source: PLoS One. 2015 Feb 13;10(2):e0118353. doi: 10.1371/journal.pone.0118353 (PMC4334511; doi:10.1371/journal.pone.0118353)
Supplement: S5 Table — (DOCX) [file pone.0118353.s006.docx]

**S5 Table: Distribution choices for the probabilistic sensitivity analysis.**

| **Input** | **Statistical Distribution** | **Ersatz Function** |
| --- | --- | --- |
| Salt | Pert (best, min, max) | ErPert = (mean, lower and upper confidence interval values) |
| Saturated Fat | Pert (best, min, max) | ErPert = (mean, lower and upper confidence interval values) |
| Trans-fat | Normal (best, min, max) | ErNormal = (mean, standard deviation) |
| Number of deaths | Poisson (count) | ErPoisson= (count) |
